# ﻿A new hexactinellid-sponge-associated zoantharian (Porifera, Hexasterophora) from the northwestern Pacific Ocean

**DOI:** 10.3897/zookeys.1156.96698

**Published:** 2023-03-24

**Authors:** Hiroki Kise, Miyuki Nishijima, Akira Iguchi, Junpei Minatoya, Hiroyuki Yokooka, Yuji Ise, Atsushi Suzuki

**Affiliations:** 1 Geological Survey of Japan, National Institute of Advanced Industrial Science and Technology (AIST), AIST Tsukuba Central 7, 1-1-1 Higashi, Tsukuba, Ibaraki 305-8567, Japan Geological Survey of Japan, National Institute of Advanced Industrial Science and Technology (AIST) Tsukuba Japan; 2 Research Laboratory on Environmentally-conscious Developments and Technologies (E-code), National Institute of Advanced Industrial Science and Technology (AIST), Tsukuba 305-8567, Japan Research Laboratory on Environmentally-conscious Developments and Technologies (E-code), National Institute of Advanced Industrial Science and Technology (AIST) Tsukuba Japan; 3 Japan Organization for Metals and Energy Security (JOGMEC), 2-10-1 Minato-ku, Tokyo 105-0001, Japan Japan Organization for Metals and Energy Security Minato-ku Japan; 4 Institute of Environmental Ecology, IDEA Consultants, Inc., 1334-5 Riemon, Yaizu-shi, Shizuoka 421-0212, Japan Institute of Environmental Ecology, IDEA Consultants, Inc. Yaizu Japan; 5 Kuroshio Biological Research Foundation, 560 Nishidomari, Otsuki, Hata, Kochi 788-0333, Japan Kuroshio Biological Research Foundation Otsuki Japan

**Keywords:** Glass sponge, Hexasterophora, host specificity, molecular phylogeny, symbiosis

## Abstract

Symbiotic associations between zoantharians and sponges can be divided into two groups: those that associate with Demospongiae and those that associate with Hexactinellida. *Parachurabanashinseimaruae* Kise, **gen. nov. et sp. nov.**, a new genus and a new species of Hexactinellida-associated zoantharian from Japanese waters, is described. It is characterized by a combination of the following: i) its host hexactinellid sponge, ii) very flat polyps, iii) cteniform endodermal marginal muscles, and iv) characteristic mutations in three mitochondrial regions (including a unique 26-bp deletion in 16S ribosomal DNA) and three nuclear regions. *Parachurabanashinseimaruae* Kise, **gen. nov. et sp. nov.** is the third genus in the family Parazoanthidae that is reported to be associated with Hexasterophora sponges. Although specimens have so far only been collected on Takuyo-Daigo Seamount off Minami-Torishima Island in Japan, unidentified zoantharians of similar description have been reported from the waters around Australia, indicating that the species might be widespread across the Pacific.

## ﻿Introduction

The family Parazoanthidae Delage & Hérouard, 1901 comprises 16 genera and more than 50 species (Reimer and Sinniger 2022). The Parazoanthidae usually form symbiotic relationships with various benthic invertebrates, including octocorals (Cutress and Pequegnat 1960; Reimer et al. 2008; Sinniger et al. 2013; Carreiro-Silva et al. 2017), antipatharians (Sinniger et al. 2010; Kise et al. 2017), and sponges (Duchassaing de Fonbressin and Michelotti 1860; Schmidt 1862; Montenegro et al. 2015). This allows them to capture plankton more effectively in environments where plankton are scarce by attaching themselves to benthic filter feeders (Di Camillo et al. 2010).

Sinniger et al. (2005, 2010) suggested that different genera within the Parazoanthidae share evolutionary histories with their associated host organisms, as these genera form monophyletic clades based on associated host organisms. Symbiotic associations between parazoanthids and sponges can be divided into two groups: those that associate with Demospongiae and those that associate with Hexactinellida. Demospongiae-associated zoantharians consist of *Bergia* Duchassaing & Michelotti, 1860, *Parazoanthus* Haddon & Shackleton, 1891, and *Umimayanthus* Montenegro, Sinniger & Reimer, 2015; Hexactinellida-associated zoantharians comprise *Churabana* Kise, Montenegro & Reimer, 2021, *Isozoanthus* Carlgren in Chun, 1903, and *Vitrumanthus* Kise, Montenegro & Reimer, 2021. *Churabana* and *Vitrumanthus* are recently established genera that are characterized by their association with the hexactinellid subclass Hexasterophora (Kise et al. 2022). *Churabanakuroshioae* Kise, Montenegro & Reimer, 2021 and *Vitrumanthusoligomyarius* (Wassilieff, 1908) are found in the Pacific Ocean, and *V.schrieri* Kise, Montenegro & Reimer, 2021 and *V.vanderlandi* Kise, Montenegro & Reimer, 2021 are found in the Atlantic Ocean, including the Dutch Caribbean and the western coast of Africa. Although Hexasterophora–zoantharian associations are relatively common and have been reported to occur circumglobally, potentially undescribed zoantharians have been observed on hexasterophoran sponges such as *Cyrtauloncaledoniensis* Reiswig & Kelly, 2017 in the Pacific Ocean (Reiswig and Kelly 2017). Thus, the diversity of Hexasterophora-associated zoantharians remains understudied in this region.

Recently, we collected a single specimen of parazoanthid associated with a hexactinellid sponge in the family Farreidae Gray, 1872 during a benthic survey of the Takuyo-Daigo Seamount in the western Pacific Ocean. On the basis of molecular phylogenetic analyses combined with morphological and ecological data, we formally describe it here as the new species *Parachurabanashinseimaruae* gen. nov. et sp. nov. (authored by Kise).

## ﻿Materials and methods

### ﻿Specimen collection

A single specimen was collected on 19 June 2020 by using a remotely operated submersible on Takuyo-Daigo Seamount off southwestern Minami-Torishima Island in the northwestern Pacific Ocean during a cruise aboard the RV *Shinsei-maru*. Photographs of the specimen were taken in situ for gross external morphological observation. The collected specimen was fixed in 99.5% EtOH and stored at –80 °C.

### ﻿Molecular analyses

Genomic DNA was extracted from the tissue of the holotype specimen using a spin-column DNeasy Blood and Tissue Extraction Kit (Qiagen, Hilden, Germany) following the manufacture’s protocol. PCR amplification using Takara Ex Taq DNA Polymerase Hot Start Version (TaKaRa Bio, Inc., Shiga, Japan) was conducted for mitochondrial cytochrome c oxidase subunit I (COI) with jgLCO1490 and jgHCO2198 (Geller et al. 2013), mitochondrial 12S ribosomal DNA (mt 12S-rDNA) with the primers 12S1a and 12S3r (Sinniger et al. 2005), mitochondrial 16S ribosomal DNA (mt 16S-rDNA) with the primers 16Sant0a (Sinniger et al. 2010) and 16SbmoH (Sinniger et al. 2005), nuclear 18S ribosomal DNA (18S-rDNA) with the primers 18SA and 18SB (Medlin et al. 1988), nuclear internal transcribed spacer region of ribosomal DNA (ITS-rDNA) with the primers ITSf and ITSr (Swain 2010), and nuclear 28S ribosomal DNA (28S-rDNA) with the primers 28Sf and 28Sr (Swain 2009). For 18S-rDNA, the primers 18SC, 18SL, 18SO, and 18SY (Apakupakul et al. 1999) were used for sequencing.

All PCR products were purified with ExoSAP-IT™ PCR Product Cleanup Reagent (Thermo Fisher Scientific, Waltham, MA, USA) at 37 °C for 15 min followed by 80 °C for 15 min. Purified PCR products were sequenced by Macrogen Japan, Inc. (Kyoto, Japan). Obtained sequences in this study were deposited in GenBank (Suppl. material 1)

Bidirectional sequences were assembled and edited in Geneious v. 10.2.3 (Kearse et al. 2012). Multiple sequence alignments were performed with previously published Parazoanthidae sequences obtained from GenBank (Suppl. material 1) using MAFFT v. 7.110 (Katoh and Standley 2013) with the auto algorithm under default parameters. Epizoanthidae Delage & Hérouard, 1901 and *Isozoanthus* Carlgren in Chun, 1903 were selected as outgroups. Although *Isozoanthus* is currently located in Parazoanthidae, recent studies suggest that *Isozoanthus* is phylogenetically closer to Epizoanthidae than Parazoanthidae (e.g., Swain 2010). All aligned datasets are available at figshare (https://doi.org/10.6084/m9.figshare.21673196).

Phylogenetic analyses were performed on the concatenated dataset using maximum likelihood (ML) and Bayesian inference (BI). ModelTest-NG v. 0.1.6 (Darriba et al. 2019) and the Akaike information criterion were used to independently select the best-fitting model for each molecular marker for both ML and BI. The best models for ML and BI analyses were TrN+I+G (BI: HKY+I+G) for COI, TPM3uf+I+G (BI: HKY+G) for mt 12S-rDNA, GTR+G for mt 16S-rDNA, HKY+I+G for 18S-rDNA, TPM1uf+I+G (BI: GTR+I+G) for ITS-rDNA, and GTR+I+G for 28S-rDNA. Independent phylogenetic analyses were performed using models partitioned by region in RAxML-NG v. 0.9.0 (Kozlov et al. 2019) for ML, and MrBayes v. 3.2.6 (Ronquist and Huelsenbeck 2003) for BI. RAxML-NG was configured to use 12,345 initial seeds, search for the best tree among 100 preliminary parsimony trees, scale and automatically optimize branch length for each partition, and optimize the model parameters, with 1000 bootstrap replicates. MrBayes was configured as indicated by ModelTest-NG: 4 Markov chain Monte Carlo heated chains were run for 5,000,000 generations with the temperature of the heated chain set to 0.2. Chains were sampled every 200 generations. Burn-in was set to 1,250,000 generations, at which point the average standard deviation of split frequency was consistently below 0.01.

ITS-rDNA has been considered as a useful marker to delineate species in Zoantharia (Reimer et al. 2007). Therefore, additional ML phylogenetic analysis for ITS-rDNA was performed using PhyML v. 3.0 (Guindon et al. 2010) with the best model (GTR) inferred by Smart Model Selection (SMS) implemented in the PhyML, with 1000 bootstrap replicates.

### ﻿Morphological observations

External morphological characters of the preserved specimen were examined using in-situ images and a dissecting microscope. Internal morphological characters were examined by using histological sections; 10–15 µm serial sections were made with a microtome (LEICA RM2145; Leica, Germany) and stained with haematoxylin and eosin after decalcification with Morse solution for 48 h (1:1 vol; 20% citric acid: 50% formic acid) and desilication with 20% hydrofluoric acid for 18–24 h. Classification of marginal muscle shapes followed the scheme described by Swain et al. (2015). Cnidae analysis was conducted using undischarged nematocysts from the tentacles, columns, actinopharynxes, and mesenterial filaments of two polyps of the holotype specimen under a Nikon Eclipse80i microscope (Nikon, Tokyo). Cnidae sizes were measured using ImageJ v. 1.45 (Rasband 2012). Cnidae classification followed England (1991) and Ryland and Lancaster (2004) except for the treatment of basitrichs and microbasic b-mastigophores as mentioned by Kise and Reimer (2019). Associated hexactinellid sponges were identified based on morphology (Reiswig and Wheeler 2002a, b).

### ﻿Abbreviations

**CMNH** Coastal Branch of the Natural History Museum and Institute, Chiba, Japan;

**NSMT** National Science Museum, Tsukuba, Ibaraki, Japan;

**RMNH** Rijksmuseum van Natuurlijke Historie (now at the Naturalis Biodiversity Center), Leiden, the Netherlands;

**RUMF** Ryukyu University Museum (Fujukan), University of the Ryukyus, Okinawa, Japan.

## ﻿Results

### ﻿Taxonomic description

**Order Zoantharia Rafinesque, 1815**

**Suborder Macrocnemina Haddon & Shackleton, 1891**

#### ﻿Family Parazoanthidae Delage & Hérouard, 1901

##### ﻿Genus Parachurabana

Taxon classificationAnimaliaZoanthariaParazoanthidae

Kise gen. nov.

6E14651C-CBDD-513B-BC86-B12756059BF2

https://zoobank.org/B0C6562D-BA20-42D5-B875-E91977F3C31F

###### Type species.

*Parachurabanashinseimaruae* Kise sp. nov. by original designation.

###### Diagnosis.

Parazoanthidae with symbiotic relationship with farreid sponges. Polyp cylindrical and flat when preserved. Preserved polyps 0.5–1.0 mm in height, 0.5–3.0 mm in diameter. Azooxanthellate. Cteniform endodermal marginal muscle.

###### Remarks.

*Parachurabana* gen. nov. is differentiated from other sponge-associated parazoanthids based on a combination of host-sponge identity and morphological features. *Parachurabana* gen. nov. is easily distinguished from the genera *Bergia*, *Parazoanthus*, and *Umimayanthus* by its association with hexactinellid sponges, as the three other genera are associated with Demospongiae sponges. In Hexactinellida-sponge-associated Parazoanthidae genera, the association with subclass Amphidiscophora differentiates *Parachurabana* gen. nov. from *Isozoanthus*. Marginal muscle morphology differentiates *Parachurabana* gen. nov. (cteniform endodermal marginal muscles) from *Vitrumanthus* (cyclically transitional marginal muscles). *Parachurabana* gen. nov. can be distinguished from *Churabana* by polyp size, as *Parachurabana* gen. nov. has very flat polyps when preserved (0.5–1.0 mm in height, 0.5–3.0 mm in diameter) in comparison to *Churabana* (3.0–4.0 mm in height, 2.8–4.0 mm in diameter). In the16S-rDNA region, *Parachurabana* gen. nov. is characterized by a unique deletion of 26 bp (positions 136–150 and 168–178 in our alignment) (Suppl. material 2).

###### Etymology.

*Parachurabana* alludes to its morphological similarities to *Churabana*. The Prefix “*para*” is a Greek word meaning “resembling.”

##### ﻿ Parachurabana shinseimaruae

Taxon classificationAnimaliaZoanthariaParazoanthidae

Kise sp. nov.

6B15928E-6045-5E70-AEDB-39F12B95F89C

https://zoobank.org/908AC687-D304-4881-A097-F1BA98340F6D

Figs 1 , 2 , 3

###### Material examined.

***Holotype*.**NSMT-Co 1819, Takuyo-Daigo Seamount off southwestern Minami-Torishima Island, 23°23'N, 153°04'E, 935 m depth, coll. RV *Shinsei-maru*, 19 June 2020, fixed in 99.5% ethanol.

###### Material examined for comparison.

*Churabanakuroshioae*RUMF-ZG-04447 (holotype), collected from near Iejima Island, Motobu, Okinawa, Japan by T. Higashiji, 02 Mar. 2018. *Vitrumanthusschrieri*RMNH.COEL.42429 (holotype), collected from SubStation, Curaçao by B.W. Hoeksema, 31 Mar. 2014. *Vitrumanthusvanderlandi*RMNH.COEL.42623 (holotype), Cape Verde Islands, São Tiago, Ilheus Rombos east of Cima by RV *HNIMS Tydeman*, 24 Aug. 1986. *Vitrumanthusoligomyarius*CMNH ZG-4785, off Katsuura, Chiba, Japan by A. Tamura, 19 Jan. 2006.

###### Description.

**Figure 1.**
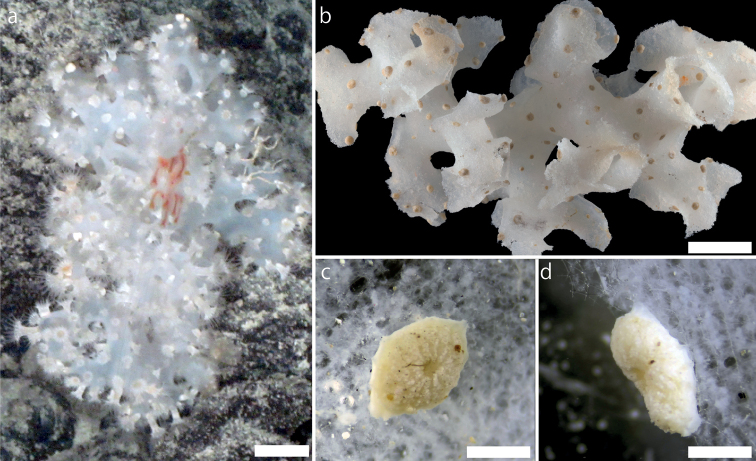
External morphology of *Parachurabanashinseimaruae* sp. nov. **a** photographic record from Takuyo-Daigo Seamount off southwestern Minami-Torishima Island **b–d**NSMT-Co 1819 **a** living polyps on a hexactinellid sponge *Farrea* sp. **b** preserved specimen **c** close-up image of a single preserved polyp **d** close-up, side-view image of a single preserved polyp attached to a hexactinellid sponge *Farrea* sp. Scale bars: 20 mm (**a, b**); 1 mm (**c, d**).

***External morphology*.** Cylindrical polyps that appear solitary and sparsely distributed on the hexactinellid sponge *Farrea* Bowerbank, 1862 (Fig. 1a, b). Surface of column rough, and ectoderm continuous. Polyps attached to hexactinellid sponge surfaces with pedal-disk-like structure (Fig. 1c, d). In contracted polyp, tentacles poorly covered by capitulum and actinopharynx visible. Preserved column creamy white in color and heavily encrusted with sand and silica particles. Capitulary ridges discernible, 12–14 in number (Fig. 1c). Tentacles 24–28 in number, shorter than or equal to expanded oral disk diameter. Living expanded polyps to ca. 10.0 mm in height and 5.0 mm in diameter. Preserved contracted polyps to 0.5–1.0 mm in height and 0.5–3.0 mm in diameter. Living column white and/or yellowish; capitulum and tentacle transparent (Fig. 1a).

**Figure 2.**
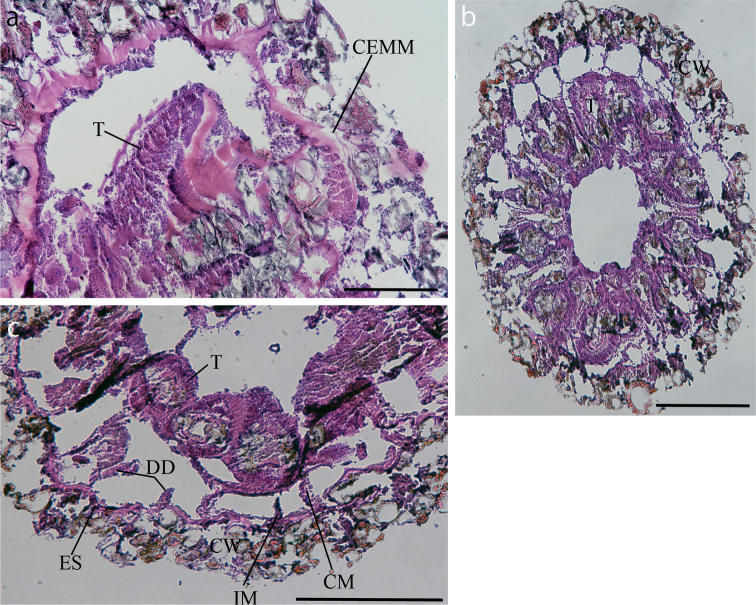
Images of the internal morphology of *Parachurabanashinseimaruae* sp. nov. NSMT-Co 1819 **a** close-up image of cteniform endodermal marginal muscle in a longitudinal polyp section **b** cross-section at the height of tentacles **c** cross-section at the height of the actinopharynx. Abbreviations: CEMM, cteniform endodermal marginal muscle; CM, complete mesentery; CW, column wall; DD, dorsal directives; ES, encircling sinus; IM, incomplete mesentery; T, tentacles. Scale bars: 200 µm (**a**); 500 µm (**b, c**).

***Internal morphology*.** Zooxanthellae absent. Cteniform endodermal marginal muscle with comb-like mesogleal pleats (Fig. 2a). Ectoderm and mesoglea heavily encrusted with numerous sand and silica particles of various size (Fig. 2b, c). Basal canals of mesenteries absent and encircling sinus visible (Fig. 2c). Single siphonoglyph and complete mesenteries possibly fertile.

**Figure 3.**
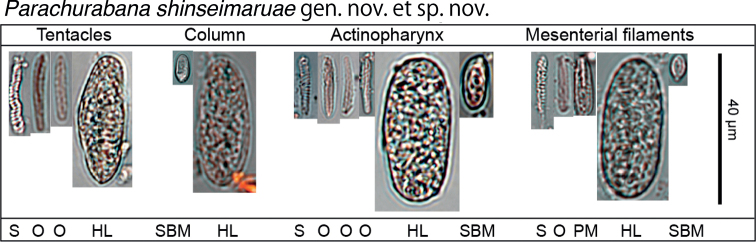
Cnidae in the tentacles, column, actinopharynx, and mesenterial filaments of the holotype of *Parachurabanashinseimaruae* sp. nov. Abbreviations: HL, holotrich large; O, basitrichs and microbasic b-mastigophores; SBM, special microbasic b-mastigophores; PM, microbasic p-mastigophores; S, spriocysts.

***Cnidae*.** Basitrichs and microbasic b-mastigophores, microbasic p-mastigophores, holotrichs, special b-mastigophores, and spirocysts (See Fig. 3 and Table 1 for size).

**Table 1.** Cnidae types and sizes observed in this study. Frequency: relative abundance of cnidae type in decreasing order; numerous, common, occasional, rare. *n* = number of cnidae measured.

Tissue	Type of cnidae	Length (min–max, mean)	Width (min–max, mean)	Frequency	*n*
Tentacle	Spirocysts	15.30–32.20, 23.40	2.22–4.81, 3.64	Numerous	195
	Basitrichs and microbasic b-mastigophores	15.31–25.12, 21.54	1.61–4.20, 3.38	Numerous	67
	Holotrichs (L)	27.97–44.58, 35.29	11.63–21.31, 15.20	Occasional	11
Column	Special microbasic b-mastigophores	11.56–16.84, 13.91	5.16–8.04, 6.01	Occasional	13
	Holotrich (L)	28.84–37.84, 32.00	10.72–18.72, 15.35	Common	18
Actinopharynx	Spirocysts	18.05–29.02, 23.73	2.03–4.62, 3.45	Numerous	45
	Basitrichs and microbasic b-mastigophores	18.29–27.64, 22.33	1.73–4.96, 3.30	Common	32
	Special microbasic b-mastigophores	17.88–19.09, 18.48	5.69–5.73, 5.71	Rare	2
	Holotrichs (L)	38.33–48.65, 43.00	11.08–17.51, 13.96	Rare	3
Mesenterial filaments	Spirocysts	19.31–32.17, 24.76	2.26–4.81, 3.40	Occasional	11
	Bastrichs and microbasic b-mastigophores	17.81–27.46, 22.45	3.38–4.57, 3.83	Common	15
	Microbasic p-mastigophores	14.04–23.25, 19.31	5.08–7.51, 5.96	Occasional	13
	Special microbasic b-mastigophores	5.84–10.71, 7.79	2.68–5.27, 4.21	Occasional	11
	Holotrichs (L)	33.13–48.86, 38.93	12.39–24.04, 16.39	Common	22

###### Distribution and habitats.

Northwestern Pacific Ocean: Takuyo-Daigo Seamount off southwestern Minami-Torishima Island at depths of 900–1000 m.

###### Associated host.

*Farrea* sp. (Porifera: Hexactinellida)

###### Molecular phylogeny.

**Figure 4.**
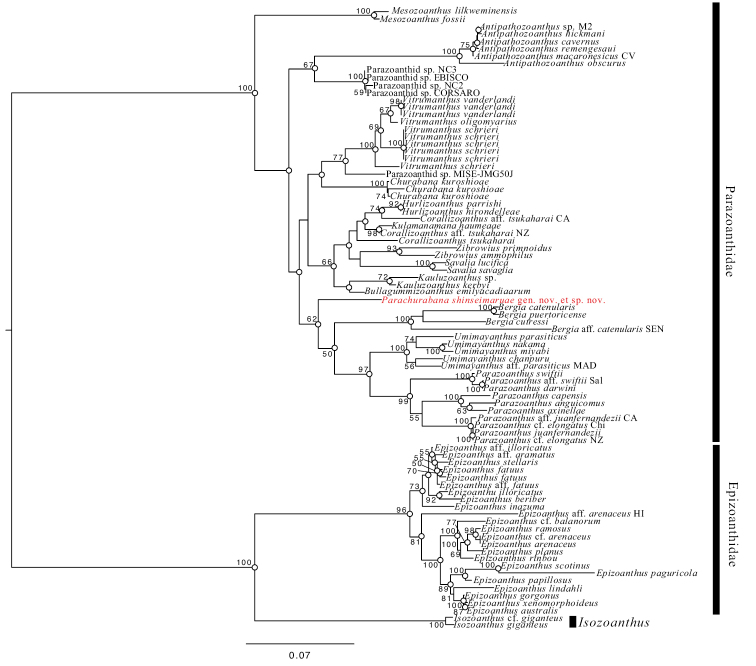
Maximum-likelihood tree based on combined dataset of COI, 12S-rDNA, 16S-rDNA, 18S-rDNA, 28S-rDNA, and ITS-rDNA sequences. Number at nodes represent ML bootstrap values (>50% are shown). White circles on nodes indicate high support of Bayesian posterior probabilities (>0.95).

Both ML and BI phylogenetic analyses using the concatenate dataset indicate that *Parachurabanashinseimaruae* sp. nov. is basal to the clade containing the genera *Bergia*, *Parazoanthus*, and *Umimayanthus* (Fig. 4; ML, 62%; BI, 0.99). ML phylogenetic analyses place *Churabana* and *Vitrumanthus* in a clade with octocoral-associated genera such as *Corallizoanthus* Reimer in Reimer, Nonaka, Sinniger & Iwase, 2008 with no support (ML < 50%), whereas BI phylogenetic analyses place *Churabana* and *Vitrumanthus* in a sister clade to that containing *Parachurabana*, *Bergia*, *Parazoanthus*, and *Umimayanthus* (Suppl. material 3). The topology of a phylogeny for ITS-rDNA dataset was similar with the concatenate dataset (Suppl. material 4).

###### Remarks.

*Parachurabanashinseimaruae* sp. nov. has so far only been identified on one seamount off southwestern Minami-Torishima Island. However, *Parachurabanashinseimaruae* sp. nov. may be distributed across the Pacific Ocean, as several specimens associated with farreid sponges have been observed in Australian waters (M. Ekins personal communication). Although *Parachurabanashinseimaruae* sp. nov. is morphologically similar to *Vitrumanthusschrieri*, *Parachurabanashinseimaruae* sp. nov. and *V.schrieri* can be separated by marginal muscle (cteniform endodermal marginal muscle vs cyclically transitional marginal muscle). Furthermore, *Parachurabanashinseimaruae* sp. nov. can be distinguished from *Churabanakuroshioae* by polyp size (0.5–1.0 mm in height by 0.5–3.0 mm in diameter vs 3.0–4.0 mm in height by 2.8–4.0 mm in diameter).

###### Etymology.

The species is named after RV *Shinsei-maru*, as the type specimens were collected by this vessel.

## ﻿Discussion

*Parachurabana* gen. nov. is the third Parazoanthidae genus known to associate with hexasterophoran sponges. Each of these three genera is associated with different hexasterophorans: *Parachurabana* gen. nov. is known to associate with *Farrea* (family Farreidae); *Churabana* with *Pararete* Ijima, 1927 (Euretidae); and *Vitrumanthus* with *Verrucocoeloidea* Reid, 1969 (Euretidae), *Cyrtaulon* Schulze, 1886 (*Sceptrulophoraincertaesedis*), *Aphrocallistes* Gray, 1858 (Aphrocallistidae), and *Tretochone* Reid, 1958 (Euretidae) (Kise et al. 2022). In addition, *Vitrumanthus* is also known to associate to *Parahigginsia* Dendy, 1924 within the Demospongiae (Kise et al. 2022). Although *Parachurabana* gen. nov. may be host specific to the genus *Farrea*, recent studies suggest that the association between zoantharians and host organisms can be more flexible than initially presumed (see Vaga et al. 2020). Further studies on more taxa are required to evaluate host specificity between zoantharians and hexasterophorans. Recent studies indicate that the deep sea harbors high levels of zoantharian diversity (e.g., Sinniger et al. 2013; Carreiro-Silva et al. 2017; Reimer et al. 2019). However, taxonomic studies on the deep-sea zoantharians are generally lacking and numerous undescribed species await formal description. This study contributes to filling this taxonomic gap.

## Supplementary Material

XML Treatment for Parachurabana

XML Treatment for Parachurabana shinseimaruae

## Supplementary materials

Supplementary material 1 GenBank accession numbers used for phylogenetic analyses in this study Data type Accession numbers https://binary.pensoft.net/file/829366This dataset is made available under the Open Database License (http://opendatacommons.org/licenses/odbl/1.0/). The Open Database License (ODbL) is a license agreement intended to allow users to freely share, modify, and use this Dataset while maintaining this same freedom for others, provided that the original source and author(s) are credited.Hiroki Kise, Miyuki Nishijima, Akira Iguchi, Junpei Minatoya, Hiroyuki Yokooka, Yuji Ise, Atsushi Suzuki

Supplementary material 2 Summary of deletions in the alignment of 16S-rDNA that characterize *Parachurabana* gen. nov. Data type figure (eps file) https://binary.pensoft.net/file/829367This dataset is made available under the Open Database License (http://opendatacommons.org/licenses/odbl/1.0/). The Open Database License (ODbL) is a license agreement intended to allow users to freely share, modify, and use this Dataset while maintaining this same freedom for others, provided that the original source and author(s) are credited.Hiroki Kise, Miyuki Nishijima, Akira Iguchi, Junpei Minatoya, Hiroyuki Yokooka, Yuji Ise, Atsushi Suzuki

Supplementary material 3 Bayesian-inference tree based on combined dataset of COI, 12S-rDNA, 16S-rDNA, 18S-rDNA, 28S-rDNA, and ITS-rDNA sequences. Number at nodes represent Bayesian posterior probabilities (>0.95) Data type figure (pdf file) https://binary.pensoft.net/file/829368This dataset is made available under the Open Database License (http://opendatacommons.org/licenses/odbl/1.0/). The Open Database License (ODbL) is a license agreement intended to allow users to freely share, modify, and use this Dataset while maintaining this same freedom for others, provided that the original source and author(s) are credited.Hiroki Kise, Miyuki Nishijima, Akira Iguchi, Junpei Minatoya, Hiroyuki Yokooka, Yuji Ise, Atsushi Suzuki

Supplementary material 4 Maximum-likelihood tree based on ITS-rDNA sequences. Number at nodes represent ML bootstrap values (>50% are shown) Data type figure (pdf file) https://binary.pensoft.net/file/829369This dataset is made available under the Open Database License (http://opendatacommons.org/licenses/odbl/1.0/). The Open Database License (ODbL) is a license agreement intended to allow users to freely share, modify, and use this Dataset while maintaining this same freedom for others, provided that the original source and author(s) are credited.Hiroki Kise, Miyuki Nishijima, Akira Iguchi, Junpei Minatoya, Hiroyuki Yokooka, Yuji Ise, Atsushi Suzuki
