# Supplementary figures and images for: ﻿A new hexactinellid-sponge-associated zoantharian (Porifera, Hexasterophora) from the northwestern Pacific Ocean
Source: Zookeys. 2023 Mar 24;1156:71–85. doi: 10.3897/zookeys.1156.96698 (PMC10208231; doi:10.3897/zookeys.1156.96698)

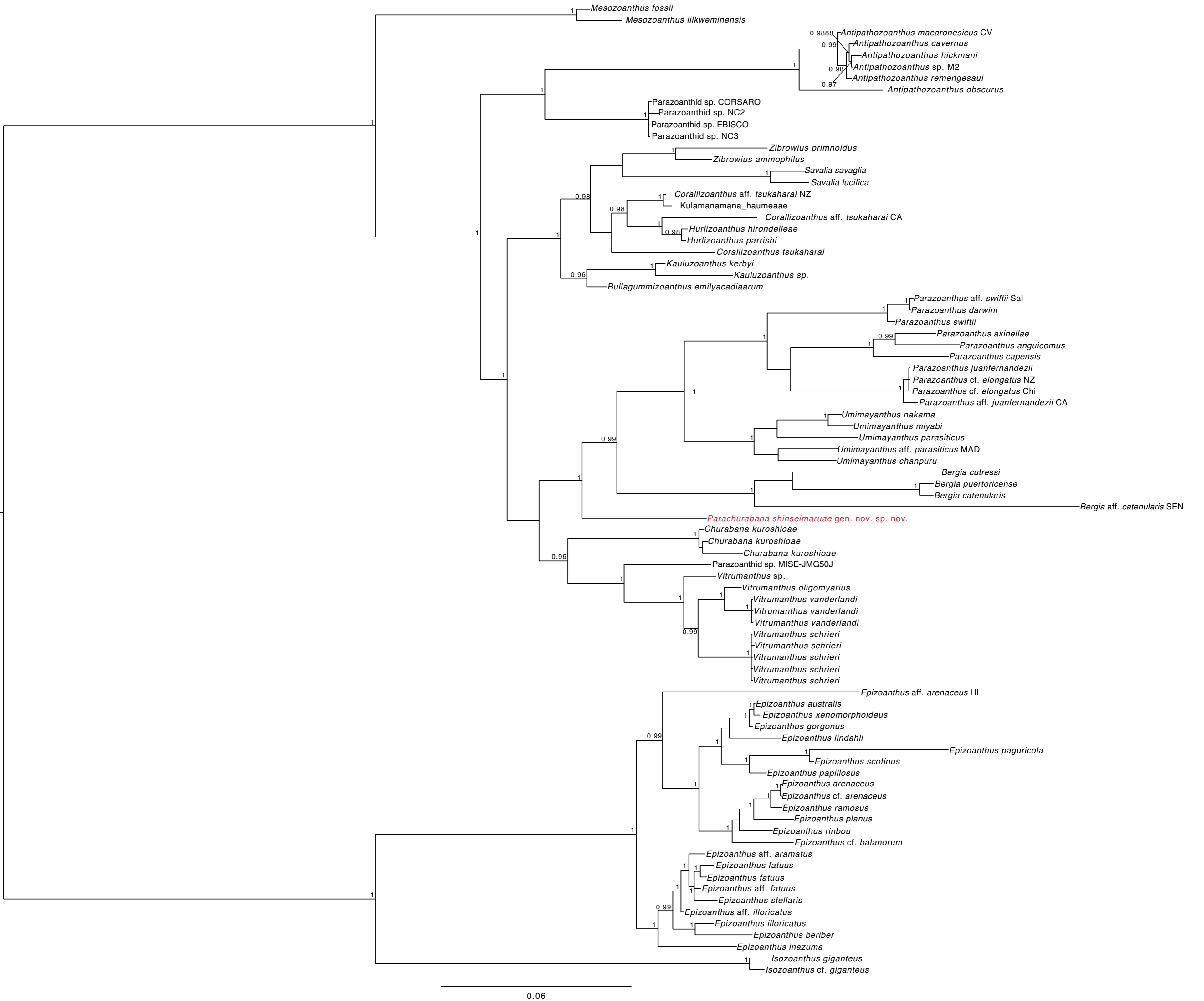

Supplement: Supplementary material 3 — Bayesian-inference tree based on combined dataset of COI, 12S-rDNA, 16S-rDNA, 18S-rDNA, 28S-rDNA, and ITS-rDNA sequences. Number at nodes represent Bayesian posterior probabilities (>0.95) [file zookeys-1156-071_article-96698__-s003.pdf]

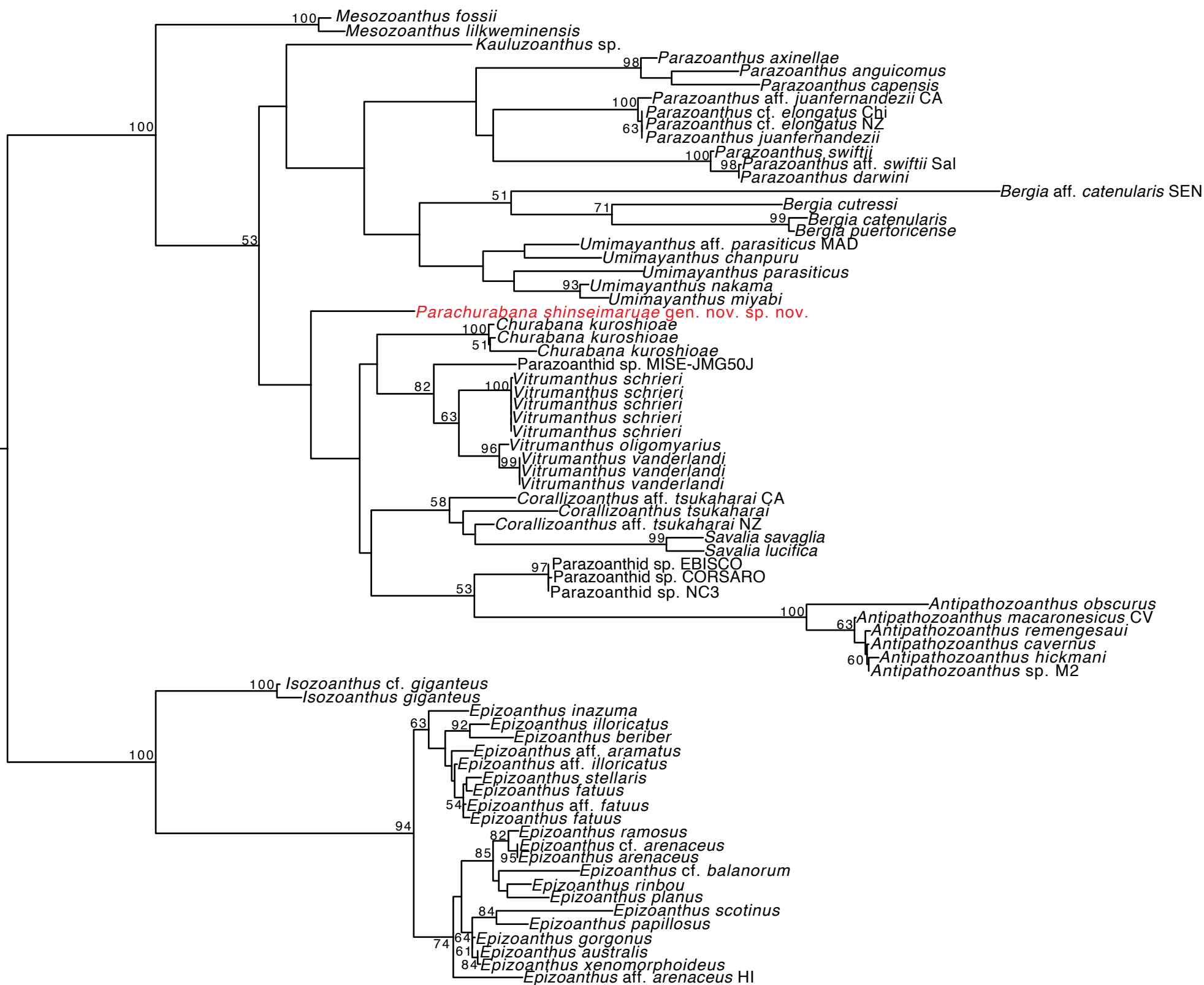

0.3

Supplement: Supplementary material 4 — Maximum-likelihood tree based on ITS-rDNA sequences. Number at nodes represent ML bootstrap values (>50% are shown) [file zookeys-1156-071_article-96698__-s004.pdf]
